# Quantum speedup, non-Markovianity and formation of bound state

**DOI:** 10.1038/s41598-019-51290-x

**Published:** 2019-10-18

**Authors:** Bahram Ahansaz, Abbas Ektesabi

**Affiliations:** Physics Department, Azarbaijan Shahid Madani University, Tabriz, Iran

**Keywords:** Quantum information, Quantum mechanics

## Abstract

In this paper, we investigate the relationship between the quantum speedup, non-Markovianity and formation of a system-environment bound state. Previous results show a monotonic relation between these three such that providing bound states with more negative energy can lead to a higher degree of non-Markovianity, and hence to a greater speed of quantum evolution. By studying dynamics of a dissipative two-level system or a V-type three-level system, when similar and additional systems are present, we reveal that the quantum speedup is exclusively related to the formation of the system-environment bound state, while the non-Markovian effect of the system dynamics is neither necessary nor sufficient to speed up the quantum evolution. On the other hand, it is shown that only the formation of the system-environment bound state plays a decisive role in the acceleration of the quantum evolution.

## Introduction

The quantum speed limit (QSL) time^1,2^ is defined as the minimal time a quantum system needs to evolve from an initial state to a target state, which can be used to characterize the maximal speed of evolution of a quantum system. It arises in tremendous areas of quantum physics and quantum information, such as nonequilibrium thermodynamics^3^, quantum metrology^4^, quantum optimal control^5^, quantum computation^6,7^, quantum communication^3,8^, and has been studied extensively due to its fundamental importance. An important question is how to exploit the available resources to achieve the highest speed of quantum evolution where the problem of determining it is relevant to deriving physical limits in a variety of contexts. In closed systems, entanglement has been taken as a resource in the speedup of quantum evolution^9–11^. Since any real system is coupled to its environment in the practical scenarios, a considerable effort has been devoted to study controlling speedup in open quantum systems^12–14^. In this regard, Deffner and Lutz showed that the dynamical acceleration in the damped Jaynes-Cummings model for a two-level system can be induced by the non-Markovian effect, and therefore results in a smaller QSL time^14^. This phenomenon has been verified in different settings^15–18^ and confirmed by the experiment in cavity quantum electrodynamics (QED) systems^19^, where an atomic beam is treated as a controllable environment for the cavity field system. Recently, the authors of ref. ^20^ showed that both the non-Markovianity and the quantum speedup are attributed to the formation of a special eigenstate, known as a bound state, which has negative eigenenergy and is separated from the other ones by an energy gap^21–24^. It was demonstrated that if the bound state in a two-level system coupled to an environment with Ohmic spectrum is established, the evolution of the system becomes non-Markovian, and this results in quantum speedup. They found a monotonic relation between them such that providing bound states with more negative energy can result in a higher degree of non-Markovianity, and this also leads to bigger speed of quantum evolution. Despite the growing body of literature on this subject, the analysis has almost exclusively focused on the simpler settings and to our knowledge, the mechanism of speedup has not been investigated in the more complex settings.

The aim of the present study is to investigate the relationship between the quantum speedup, non-Markovianity and formation of a system-environment bound state in the more complex settings. To this aim, we study the mechanism of quantum speedup for a two-level system and a V-type three-level system in the presence of similar and additional systems. We illustrated that there is a monotonic relation between the quantum speedup and the formation of a system-environment bound state. In particular, we show that the bound states with more negative energy provided by the additional systems can speed up the quantum evolution. However, it is figure out that there is not such monotonic relation between the quantum speedup and the non-Markovianity. We demonstrate that although non-Markovianity governs the quantum speedup in the absence of additional systems, it is neither necessary nor sufficient to speed up the quantum evolution when the environment becomes more complex in the presence of additional systems. On the other hand, our results suggest that only the formation of the system-environment bound state is the essential reason for the quantum speedup.

## Results

### The condition for the existence of a bound state in a system including *N* atoms

**Figure 1:**
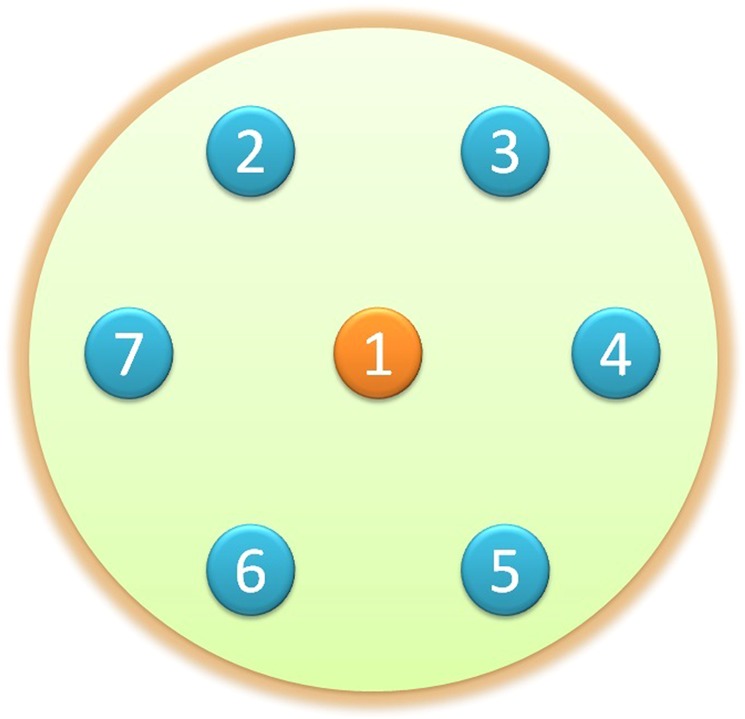
The figure corresponds to seven atoms (N = 7) immersed in a common reservoir. The central atom (the orange circle) is considered as our main concern of the single-atom system and the $N-1$ remainder ones are considered as the additional atoms (the blue circles).

This section is devoted to a system containing *N* non-interacting atoms coupled to a reservoir, as depicted in Fig. 1. As will be discussed in the following cases, we check the energy distribution of the Hamiltonian of the mentioned system by considering the atoms as two-level and three-level systems. Then, it will be shown that how the system-environment bound state, as a special eigenstate with eigenvalue residing in the band gap of the energy spectrum, can be formed in these models.

#### **Case I:**

**Two-level systems**. By considering the atoms as two-level systems, the Hamiltonian of the whole system can be written as ($\hbar =1$)

1 $$H={\omega }_{0}\,\mathop{\sum }\limits_{l=1}^{N}\,{\sigma }_{l}^{+}{\sigma }_{l}^{-}+\sum _{k}\,{\omega }_{k}{b}_{k}^{\dagger }{b}_{k}+\mathop{\sum }\limits_{l=1}^{N}\,\sum _{k}\,({g}_{k}{b}_{k}{\sigma }_{l}^{+}+{g}_{k}^{\ast }{b}_{k}^{\dagger }{\sigma }_{l}^{-}),$$

where the first term is the Hamiltonian of *N* two-level systems, the second term describes the reservoir Hamiltonian and the last term is the system-reservoir interaction Hamiltonian. Also, ${\sigma }_{l}^{+}$ $({\sigma }_{l}^{-})$ stands for the raising (lowering) operator of the *l*th atom with transition frequency ${\omega }_{0}$. ${b}_{k}^{\dagger }$ (${b}_{k}$) creates (annihilates) the *k*th field mode with frequency ${\omega }_{k}$ and the coupling strength between the *l*th atom and the *k*th field mode is represented by ${g}_{k}$.

The following eigenvalue equation gives the energy spectrum of the total Hamiltonian

2 $$H|\psi (t)\rangle =E|\psi (t)\rangle .$$

Considering the single excitation subspace, the commutation of Hamiltonian with the number operator of excitations (i.e., $[({\sum }_{l=1}^{N}\,{\sigma }_{l}^{+}{\sigma }_{l}^{-}+{\sum }_{k}\,{b}_{k}^{\dagger }{b}_{k}),H]=0$) results in the state of the whole system as follows

3 $$|\psi (t)\rangle ={\alpha }_{0}(t)|0{\rangle }_{S}|0{\rangle }_{E}+\mathop{\sum }\limits_{l=1}^{N}\,{\alpha }_{l}(t)|l{\rangle }_{S}|0{\rangle }_{E}+\sum _{k}\,{\beta }_{k}(t)|0{\rangle }_{S}|{1}_{k}{\rangle }_{E},$$

where $|l{\rangle }_{S}=|g{\rangle }_{l{\rm{th}}\equiv e}^{\otimes N}$ means that except for the *l*th atom which is excited, all other atoms are in their corresponding ground states. $|g\rangle$ and $|e\rangle$ hold for ground and excited states, respectively, and $|0{\rangle }_{S}$ is defined as $|0{\rangle }_{S}=|g{\rangle }^{\otimes N}=|g,g,\ldots ,g\rangle$. The vacuum state of the reservoir is denoted by $|0{\rangle }_{E}$, and $|{1}_{k}{\rangle }_{E}$ is the *k*th field mode with just one excitation. Now we substitute Eqs (1) and (3) into Eq. (2) and obtain the following set of $N+1$ equations

4 $$\begin{array}{rcl}{\omega }_{k}{\beta }_{k}(t)+\mathop{\sum }\limits_{l=1}^{N}\,{g}_{k}^{\ast }{\alpha }_{l}(t) & = & E{\beta }_{k}(t),\\ {\omega }_{0}{\alpha }_{j}(t)+\sum _{k}\,{g}_{k}{\beta }_{k}(t) & = & E{\alpha }_{j}(t),\,j=1,2,\ldots ,N.\end{array}$$

If one obtain ${\beta }_{k}(t)$ from the first equation and substitute it into the rest of the equations, the following *N* integro-differential equations are obtained

5 $$(E-{\omega }_{0}){\alpha }_{j}(t)=-\,{\int }_{0}^{\infty }\,\frac{J(\omega )d\omega }{\omega -E}\,\mathop{\sum }\limits_{l=1}^{N}\,{\alpha }_{l}(t),\,j=1,2,\ldots ,N.$$

After summing up the Eq. (5) and eliminating ${\sum }_{l=1}^{N}\,{\alpha }_{l}(t)$ from both sides, we can obtain

6 $${\mathscr{K}}(E)=E,$$

with

7 $${\mathscr{K}}(E)={\omega }_{0}-N\,{\int }_{0}^{\infty }\,\frac{J(\omega )d\omega }{\omega -E},$$

where depending on the functionality of the spectral density $J(\omega )$ of the reservoir and the number of atoms $N$, we will have different results. In a quantum many-body system, the bound state is defined as an eigenstate with real (discrete) eigenvalue. So, it can be claimed that the system possesses a bound state, when Eq. (6) has a real root. It can be easily seen that ${\mathscr{K}}(E)$ decreases monotonically with the increase of $E$ in the regime of $E < 0$. As a result, when the condition ${\mathscr{K}}(0) < 0$ is satisfied, the function on the right-hand side of Eq. (6) always has one and only one intersection with ${\mathscr{K}}(E)$. This root just corresponds to the eigenvalue of the formed bound state in the Hilbert space of the whole system (*N* two-level system plus its common reservoir). Moreover, we can observe that ${\mathscr{K}}(E)$ is divergent in the positive energy range, i.e. $E > 0$, and thus Eq. (7) does not have a real root to make it well defined. Therefore, no real root can be found in this range to support the existence of a further bound state.

#### **Case II:**

**Three-level systems**. After studying the energy spectrum of the system including *N* two-level systems in a common reservoir, we investigate this problem by considering three-level systems in V configuration. By considering two excited states for each atom, on the other hand, each of them spontaneously decays into the ground state in such a way that the respective dipole moments of transitions may have interaction with each other through spontaneously generated interference (SGI). Also, for a given V-type atom, the decaying frequencies of excited states $|A\rangle$ and $|B\rangle$ into the ground state $|C\rangle$ are denoted by ${\omega }_{A}$ and ${\omega }_{B}$, respectively. The Hamiltonian for this described system can be written as

8 $$H^{\prime} =\mathop{\sum }\limits_{l=1}^{N}\,\sum _{m=A,B}\,{\omega }_{m}{\sigma }_{m}^{l+}{\sigma }_{m}^{l-}+\sum _{k}\,{\omega }_{k}{b}_{k}^{\dagger }{b}_{k}+\mathop{\sum }\limits_{l=1}^{N}\,\sum _{m=A,B}\,\sum _{k}\,({g}_{mk}{\sigma }_{m}^{l+}{b}_{k}+{g}_{mk}^{\ast }{\sigma }_{m}^{l-}{b}_{k}^{\dagger }).$$

The raising and lowering of the *m*th excited state of the *l*th atom to the ground state are carried out by ${\sigma }_{m}^{l\pm }(m=A,B)$. Also, ${b}_{k}$ (${b}_{k}^{\dagger }$) annihilates (creates) the *k*th field mode with the frequency ${\omega }_{k}$. Furthermore, it is assumed that ${g}_{mk}$, as the coupling strength between the *m*th excited state and the *k*th field mode, is identical for all atoms.

Now, we want to study the energy spectrum of *N* three-level systems in a common reservoir. For this purpose, the following eigenvalue equation is solved

9 $$H^{\prime} |\varphi (t)\rangle =E^{\prime} |\varphi (t)\rangle ,$$

where

10 $$\begin{array}{rcl}|\varphi (t)\rangle  & = & {\nu }_{0}(t)|0{\rangle }_{S}\otimes |0{\rangle }_{E}+\mathop{\sum }\limits_{l=1}^{N}\,({\nu }_{l}^{A}(t)|{A}_{l}\rangle +{\nu }_{l}^{B}(t)|{B}_{l}\rangle {)}_{S}\\  &  & \otimes \,|0{\rangle }_{E}+\sum _{k}\,{\eta }_{k}(t)|0{\rangle }_{S}|{1}_{k}{\rangle }_{E},\end{array}$$

is the state of the whole system in the single excitation subspace, because the total Hamiltonian conserves the number of excitations in the system, i.e. $[{\sum }_{l=1}^{N}\,({\sigma }_{A}^{l+}{\sigma }_{A}^{l-}+{\sigma }_{B}^{l+}{\sigma }_{B}^{l-})+{\sum }_{k}\,{b}_{k}^{\dagger }{b}_{k},H^{\prime} ]=0$. Also, $|0{\rangle }_{S}=|C{\rangle }^{\otimes N}$ means that all atoms are in the ground states. Moreover, $|{A}_{l}{\rangle }_{S}$ and $|{B}_{l}{\rangle }_{S}$ means that except for the *l*th atom, all other atoms are in the ground state. It is also considered that $|{A}_{l}{\rangle }_{S}$ and $|{B}_{l}{\rangle }_{S}$ are respectively in the first and second upper excited levels. We denote $|0{\rangle }_{E}$ is the vacuum state of the reservoir and $|{1}_{k}{\rangle }_{E}$ the state of it with only one excitation in the *k*th field mode. For simplicity, we assume the case of the degenerate excited levels where ${\omega }_{A}={\omega }_{B}={\omega }_{0}$. Then, by substituting Eqs (8) and (10) into Eq. (9), we obtain the following differential equations

11 $$\begin{array}{l}{\omega }_{k}{\eta }_{k}(t)+\mathop{\sum }\limits_{l=1}^{N}\,({g}_{Ak}^{\ast }{\nu }_{l}^{A}(t)+{g}_{Bk}^{\ast }{\nu }_{l}^{B}(t))=E^{\prime} {\eta }_{k}(t),\\ {\omega }_{0}{\nu }_{l}^{A}(t)+\sum _{k}\,{g}_{Ak}{\eta }_{k}(t)=E^{\prime} {\nu }_{l}^{A}(t),\,l=1,2,\ldots ,N,\\ {\omega }_{0}{\nu }_{l}^{B}(t)+\sum _{k}\,{g}_{Bk}=E^{\prime} {\nu }_{l}^{B}(t),\,l=1,2,\ldots ,N.\end{array}$$

We derive ${\eta }_{k}(t)$ from the first equation and substitute it into the rest ones, so we have

12 $$\begin{array}{rcl}(E^{\prime} -{\omega }_{0}){\nu }_{l}^{A}(t) & = & -\,{\int }_{0}^{\infty }\,\frac{J(\omega )d\omega }{\omega -E^{\prime} }\,\mathop{\sum }\limits_{l=1}^{N}\,{\nu }_{l}^{A}(t)-{\int }_{0}^{\infty }\,\frac{J^{\prime} (\omega )d\omega }{\omega -E^{\prime} }\,\mathop{\sum }\limits_{l=1}^{N}\,{\nu }_{l}^{B}(t),\\ (E^{\prime} -{\omega }_{0}){\nu }_{l}^{B}(t) & = & -\,{\int }_{0}^{\infty }\,\frac{J^{\prime} (\omega )d\omega }{\omega -E^{\prime} }\,\mathop{\sum }\limits_{l=1}^{N}\,{\nu }_{l}^{A}(t)-{\int }_{0}^{\infty }\,\frac{J(\omega )d\omega }{\omega -E^{\prime} }\,\mathop{\sum }\limits_{l=1}^{N}\,{\nu }_{l}^{B}(t).\end{array}$$

By taking a Lorentzian spectral density as given in Eq. (40) and under the assumption considered in Methods, we have $J^{\prime} (\omega )=\theta J(\omega )$ where $\theta$ is the angle between two dipole moments of the two possible transitions in each atom (see Methods). Thus, by summing up the above $2N$ equations in Eq. (12) and eliminating ${\sum }_{l=1}^{N}\,({\nu }_{l}^{A}(t)+{\nu }_{l}^{B}(t))$ from both sides, a compact form of Eq. (12) can be obtained as

13 $${\mathscr{K}}^{\prime} (E^{\prime} )=E^{\prime} ,$$

where

14 $${\mathscr{K}}^{\prime} (E^{\prime} )={\omega }_{0}-N(1+\theta )\,{\int }_{0}^{\infty }\,\frac{J(\omega )d\omega }{\omega -E^{\prime} }.$$

It is clear that the functionality of the spectral density of the reservoir $J(\omega )$, the number of atoms $N$, and the SGI parameter $\theta$ are effective factors for the solutions of Eq. (13). Nevertheless, as previously discussed in case I, the bound state is appears if Eq. (13) has at least one real solution for negative energies, i.e., $E < 0$. Otherwise, the bound state does not formed.

### QSL time and non-Markovianity

Here, we aim to evaluate QSL time bound and non-Markovianity of a single atom (two-level or three-level) in the presence of ($N-1$) additional atoms where all of the atoms are contained in a common reservoir. In this regard, for discussed cases I and II, the first atom (*l* = 1) is taken as the main concern of the our single system and ($N-1$) remainder atoms are taken as additional atoms.

According to ref.^14^, a unified expression for the QSL time is provided in open quantum systems as

15 $${\tau }_{{\rm{QSL}}}=\,{\rm{\max }}\{\frac{1}{{\Lambda }_{\tau }^{1}},\frac{1}{{\Lambda }_{\tau }^{2}},\frac{1}{{\Lambda }_{\tau }^{\infty }}\}\,{\sin }^{2}[ {\mathcal B} (\rho (0),\rho (\tau ))],$$

where ${\mathcal B} (\rho (0),\rho (\tau ))=\arccos (\sqrt{\langle \phi (0)|\rho (\tau )|\phi (0)\rangle })$, is the Bures angle between initial pure state $\rho (0)=|\phi (0)\rangle \langle \phi (0)|$ and the target state $\rho (\tau )$ governed by the time-dependent non-unitary equation ${ {\mathcal L} }_{t}(\rho (t))=\dot{\rho }(t)$, and

16 $${\Lambda }_{\tau }^{p}=\frac{1}{\tau }\,{\int }_{0}^{\tau }\,dt\parallel { {\mathcal L} }_{t}(\rho (t)){\parallel }_{p},$$

with $\parallel X{\parallel }_{p}={({x}_{1}^{p}+\ldots +{x}_{n}^{p})}^{1/p}$, which denotes the Schatten p-norm and ${x}_{1},\ldots ,{x}_{n}$ are the singular values of *X*. The intrinsic speed of a dynamical evolution is likely to evaluate by applying the recent achieved bound for the QSL time and a given actual driving time $\tau$. For the case of ${\tau }_{{\rm{QSL}}}=\tau$, there is no potential capacity for further speedup and speedup does not appear. But, when we have ${\tau }_{{\rm{QSL}}} < \tau$, it indicates the potential capacity for quantum dynamical speedup.

We first consider the exactly solvable model for a two-level system in the presence of (*N* − 1) additional atoms which are immersed in a common reservoir (see Methods). For simplicity and without loss of generality, we suppose that our considered system (the 1th atom) is initially in the excited state $\rho (0)=|1\rangle \langle 1|$ and (*N* − 1) additional atoms are in the ground state and the environment is in a vacuum state^14^. Thus, the evolved state at time *t* can be obtained as $\rho (t)=|{\alpha }_{1}(t){|}^{2}|1\rangle \langle 1|+(1-|{\alpha }_{1}(t){|}^{2})|0\rangle \langle 0|$, where the explicit form of ${\alpha }_{1}(t)$ is calculated by taking a Lorentzian spectral density for the reservoir in the Methods section. So, the QSL time bound for the considered atom can be evaluated as^17^

17 $${\tau }_{{\rm{QSL}}}=\frac{\tau (1-|{\alpha }_{1}(\tau ){|}^{2})}{{\int }_{0}^{\tau }\,|{\partial }_{t}|{\alpha }_{1}(t){|}^{2}|dt}.$$

Moreover, according to ref.^25^ the non-Markovian dynamics of the considered two-level atom can be studied through the notion of the reverse flow of information from the reservoir back to the system. Here, we apply this measure of non-Markovianity as follows

18 $$\Re =ma{x}_{{\rho }_{1,2}(0)}\,\mathop{\int }\limits_{{\vartheta } > 0}\,\,{\vartheta }[t,{\rho }_{1,2}(0)]dt,$$

where

19 $${\vartheta }[t,{\rho }_{1,2}(0)]=\frac{d}{dt}D[{\rho }_{1}(t),{\rho }_{2}(t)],$$

and $D[{\rho }_{1}(t),{\rho }_{2}(t)]=\frac{1}{2}Tr|{\rho }_{1}(t)-{\rho }_{2}(t)|$ implies the trace distance of a pair of states. The trace norm of operator $\chi$ is defined as $|\chi |=\sqrt{{\chi }^{\dagger }\chi }$. Hence, $\vartheta [t,{\rho }_{1,2}(0)]$ stands for the changing rate of the trace distance $D[{\rho }_{1}(t),{\rho }_{2}(t)]$. For our two-level system, by choosing the optimal pair of initial states obtained in ref.^26^, it is easy to check that the trace distance of the evolved states can be written as $D[{\rho }_{1}(t),{\rho }_{2}(t)]=|{\alpha }_{1}(t){|}^{2}$. Then, one can easily verify that^17^

20 $$\Re =\frac{1}{2}[|{\alpha }_{1}(\tau ){|}^{2}-1+{\int }_{0}^{\tau }\,|{\partial }_{t}|{\alpha }_{1}(t){|}^{2}|dt],$$

which connects to Eq. (17) as

21 $${\tau }_{{\rm{QSL}}}=\frac{\tau }{2\frac{\Re }{1-|{\alpha }_{1}(\tau ){|}^{2}}+1}.$$

It is clear that, the QSL time is related to the non-Markovianity $\Re$ within the driving time and the atomic excited population $|{\alpha }_{1}(\tau ){|}^{2}$.

Next, the QSL time bound for a V-type three-level atom in the presence of ($$N-1$$) atoms within a common reservoir will be obtained. To this aim, we consider the exactly solvable model for a V-type three-level atom in the presence of ($$N-1$$) additional atoms which are immersed in a common reservoir (see Methods) and assume the initial condition as $${\nu }_{1}^{A}(0)={\nu }_{1}^{B}(0)={\nu }_{1}(0)={2}^{-1/2}$$ and $${\nu }_{j}^{A}(0)={\nu }_{j}^{B}(0)={\nu }_{0}=0$$ for $$j=2,3,\ldots ,N$$, which implies that $${\nu }_{1}^{A}(t)={\nu }_{1}^{B}(t)={\nu }_{1}(t)$$. Under these assumptions, it can be easily checked that $$\parallel { {\mathcal L} }_{t}(\rho (t)){\parallel }_{\infty }=\parallel { {\mathcal L} }_{t}(\rho (t)){\parallel }_{1}$$$$/2=\parallel { {\mathcal L} }_{t}(\rho (t)){\parallel }_{2}/\sqrt{2}=2|{\partial }_{t}|{\nu }_{1}(t){|}^{2}|$$, so the following inequality holds $${\Lambda }_{\tau }^{\infty }\le {\Lambda }_{\tau }^{2}\le {\Lambda }_{\tau }^{1}$$. Also, it is clear that $${\sin }^{2}[ {\mathcal B} (\rho (0),\rho (\tau ))]=1-{\cos }^{2}[ {\mathcal B} (\rho (0),\rho (\tau ))]=1-2|{\nu }_{1}(\tau ){|}^{2}$$. Consequently, $${\tau }_{{\rm{QSL}}}$$ can be evaluated as

22 $${\tau }_{{\rm{QSL}}}=\frac{\tau (1-2|{\nu }_{1}(\tau ){|}^{2})}{2\,{\int }_{0}^{\tau }\,|{\partial }_{t}|{\nu }_{1}(t){|}^{2}|dt}.$$

Moreover, the measure of non-Markovianty for the considered V-type three-level atom can be obtained by considering the optimized pair of initial states proposed by ref.^27^. In this regard, we use the definition of trace distance, $D[{\rho }_{1}(t),{\rho }_{2}(t)]={\sum }_{l=1}^{3}\,|{\lambda }_{l}(t)|/2$, where ${\lambda }_{1}(t)=2{\nu }_{1}(t)$, ${\lambda }_{2}(t)=0$ and ${\lambda }_{3}(t)=-\,2{\nu }_{1}(t)$ are the eigenvalues of ${\rho }_{1}(t)-{\rho }_{2}(t)$. Thus, the measure of non-Markovianty in Eq. (18) is reduced to

23 $$\Re =2\,\mathop{\int }\limits_{{\partial }_{t}|{\nu }_{1}(t)| > 0}\,{\partial }_{t}|{\nu }_{1}(t)|dt.$$

Here, in contrast to the previous case, we emphasize that no explicit relationship can be established between the non-Markovianity and the QSL time bound of the considered V-type three-level atom.

## Discussion

**Figure 2:**
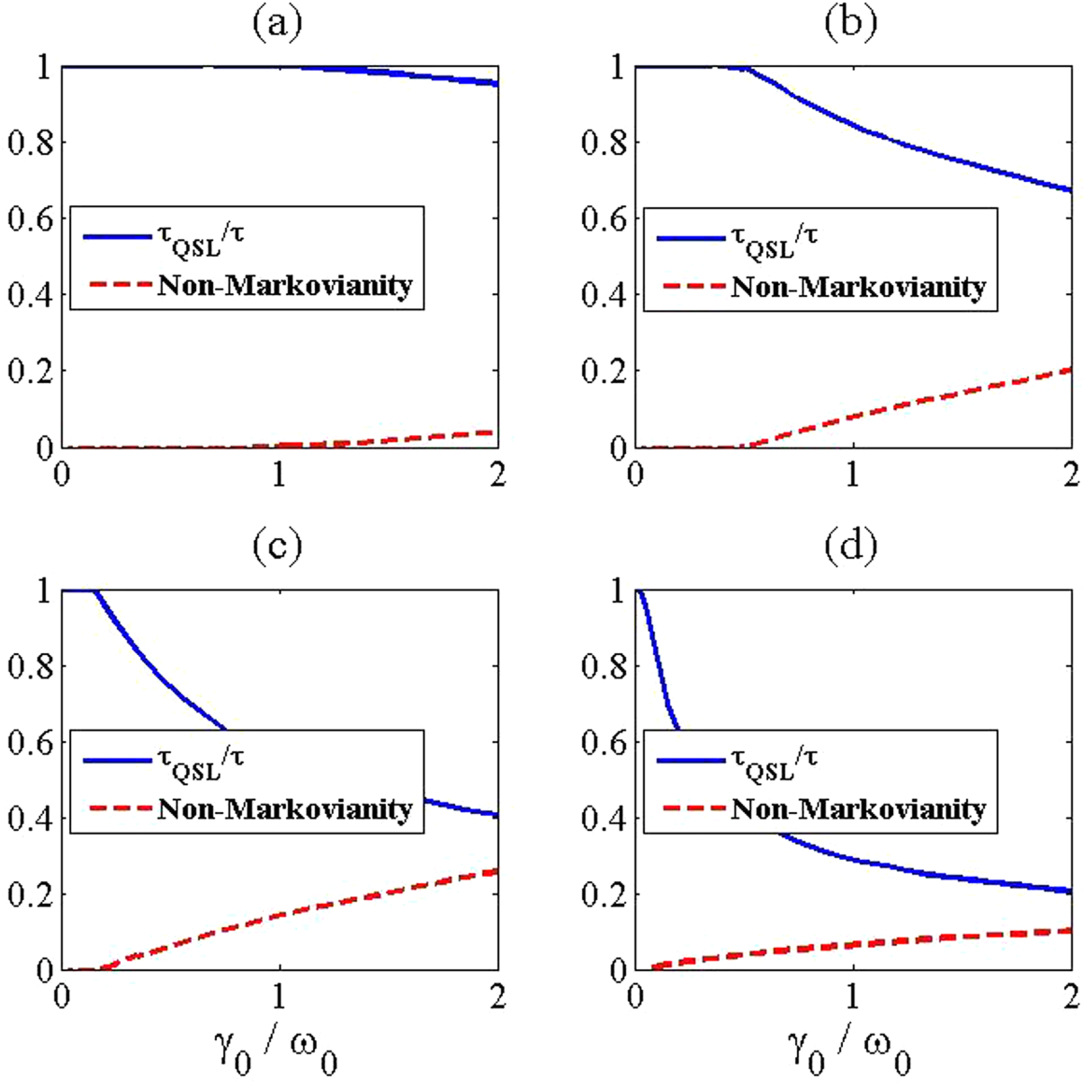
The QSL time (blue solid line) and non-Markovianity (red dashed line) for a two level system in terms of the coupling strength ${\gamma }_{0}/{\omega }_{0}$ and with different numbers of atoms in the reservoir as (**a**) *N* = 1, (**b**) *N* = 3, (**c**) *N* = 8 and (**d**) *N* = 30. The values of the used parameters are $\lambda =2$ (in units of ${\omega }_{0}$) and $\tau =5$ (in units of ${\omega }_{0}^{-1}$).

**Figure 3:**
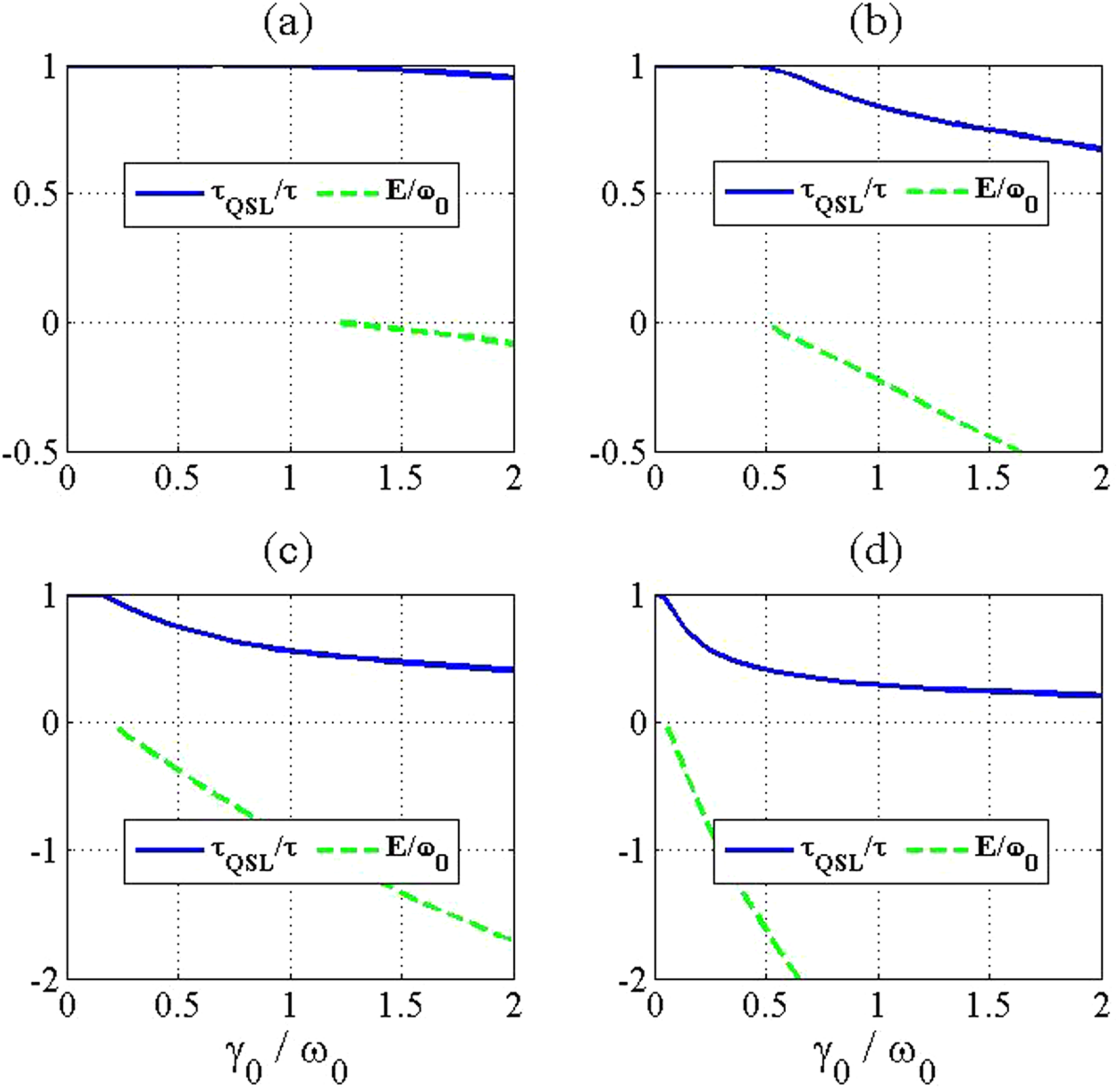
The QSL time (blue solid line) for a two level system and the energy of the formed bound state (green dashed line) in terms of the coupling strength ${\gamma }_{0}/{\omega }_{0}$ and with different numbers of atoms in the reservoir as (**a**) *N* = 1, (**b**) *N* = 3, (**c**) *N* = 8 and (**d**) *N* = 30. The values of the used parameters are $\lambda =2$ (in units of ${\omega }_{0}$) and $\tau =5$ (in units of ${\omega }_{0}^{-1}$).

In the following, we illustrate the influence of non-Markovianity and formation of a bound state on the QSL time for our considered model in the last section. In Fig. 2, we present the ratio ${\tau }_{{\rm{QSL}}}/\tau$ with the behaviors of non-Markovianity of a two-level atom in terms of the coupling strength ${\gamma }_{0}/{\omega }_{0}$ and for different numbers of additional atoms in the reservoir. Here, the ratio ${\tau }_{{\rm{QSL}}}/\tau  < 1$ shows the potential capacity for quantum dynamical speedup. As we can see in Fig. 2, in the critical point from no-speedup to speedup of quantum evolution, the Markovian environment becomes non-Markovian. Also, it is surprisingly seen that although increasing the number of additional atoms leads to more decrement of the QSL time, the non-Markovianity of the system does not always increase by inserting the additional atoms into the reservoir. This means that, non-Markovianity which is induced by the memory effect of environment cannot always induce dynamical acceleration in our considered model. To justify this, according to Eq. (21), it should be noted that when $\Re =0$ and provided that $|{\alpha }_{1}(\tau ){|}^{2}\ne 1$, no speedup can be observed in the quantum evolution, i.e. ${\tau }_{{\rm{QSL}}}=\tau$. Moreover, in the absence of additional atoms ($N=1$), and by choosing the actual driving time large enough, no population will be trapped in the excited state of the considered two-level system, i.e. $|{\alpha }_{1}(\tau ){|}^{2}\to 0$. Therefore, the QSL time will be inversely proportional to the non-Markovianity as ${\tau }_{{\rm{QSL}}}/\tau ={(2\Re +1)}^{-1}$, and as a result, the unique reason for speeding up of quantum evolution is the non-Markovianity and it induces dynamical acceleration which is matched with results in ref.^14^. However, by inserting the additional atoms into the reservoir ($N > 1$), the excited-state population approaches to a non-zero steady value in a long enough time, i.e. $|{\alpha }_{1}(\tau ){|}^{2}\to (N-1)/N$ (see Eq. (32)). Therefore, the competition between the population, $|{\alpha }_{1}(\tau ){|}^{2}$, and non-Markovianity, $\Re$, takes responsibility for the intrinsic speedup of quantum evolution. On the other hand, we can claim that the main reason for the speedup is not solely due to the non-Markovianity. One question naturally arises about an essential reflection to the quantum speedup. To answer this question, we further investigate the speedup from the perspective of formation of a system-environment bound state and demonstrate that how the quantum speedup is exclusively related to the formation of the system-environment bound state. The system-environment bound state is the stationary state of the whole system in which the inhibition of spontaneous emission is the result of the formation of it^28,29^. To this aim, the ratio ${\tau }_{{\rm{QSL}}}/\tau$ with the negative energy spectrum of the total Hamiltonian in Eq. (1), has been sketched in Fig. 3. It is obvious that the transition from no-speedup to speedup of quantum evolution is occurred faster by increasing the number of additional systems. Also, the critical points for forming the bound state match well with the ones for presenting quantum speedup. It is worth noting that providing bound states with more negative energy in the system-environment spectrum via inserting more additional atoms into the reservoir, can lead to greater speed of quantum evolution. Thus, we can conclude that the essential reason for the acceleration of evolution is related to the formation of a bound state.

**Figure 4:**
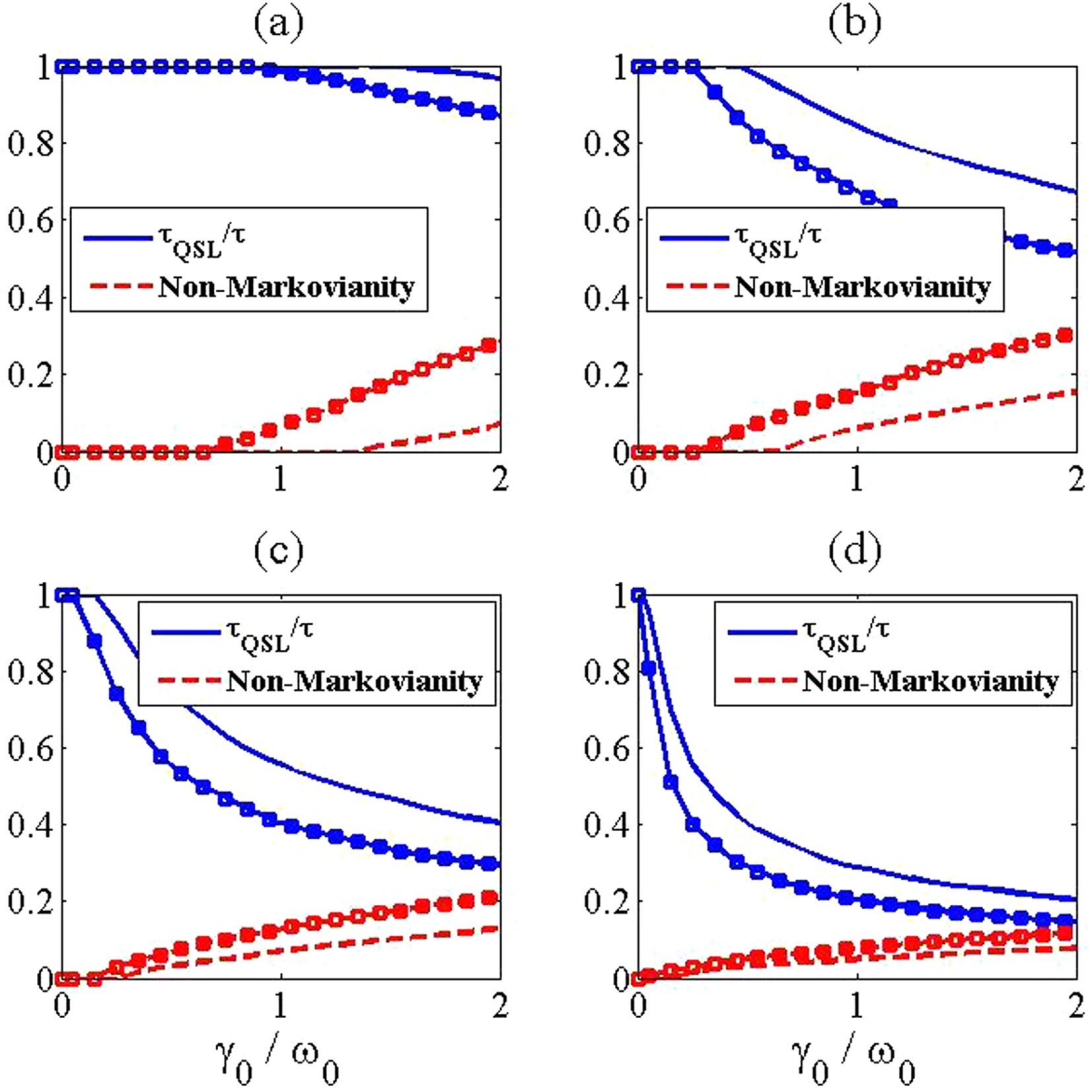
The QSL time (blue solid line) and non-Markovianity (red dashed line) for a V-type three level system in terms of the coupling strength ${\gamma }_{0}/{\omega }_{0}$ and with different numbers of atoms in the reservoir as (**a**) *N* = 1, (**b**) *N* = 3, (**c**) *N* = 8 and (**d**) *N* = 30. The values of the used parameters are $\lambda =2$ (in units of ${\omega }_{0}$) and $\tau =5$ (in units of ${\omega }_{0}^{-1}$). Also, lines with the square marks are plotted for *θ* = 1 and without the marks are plotted for *θ* = 0.

**Figure 5:**
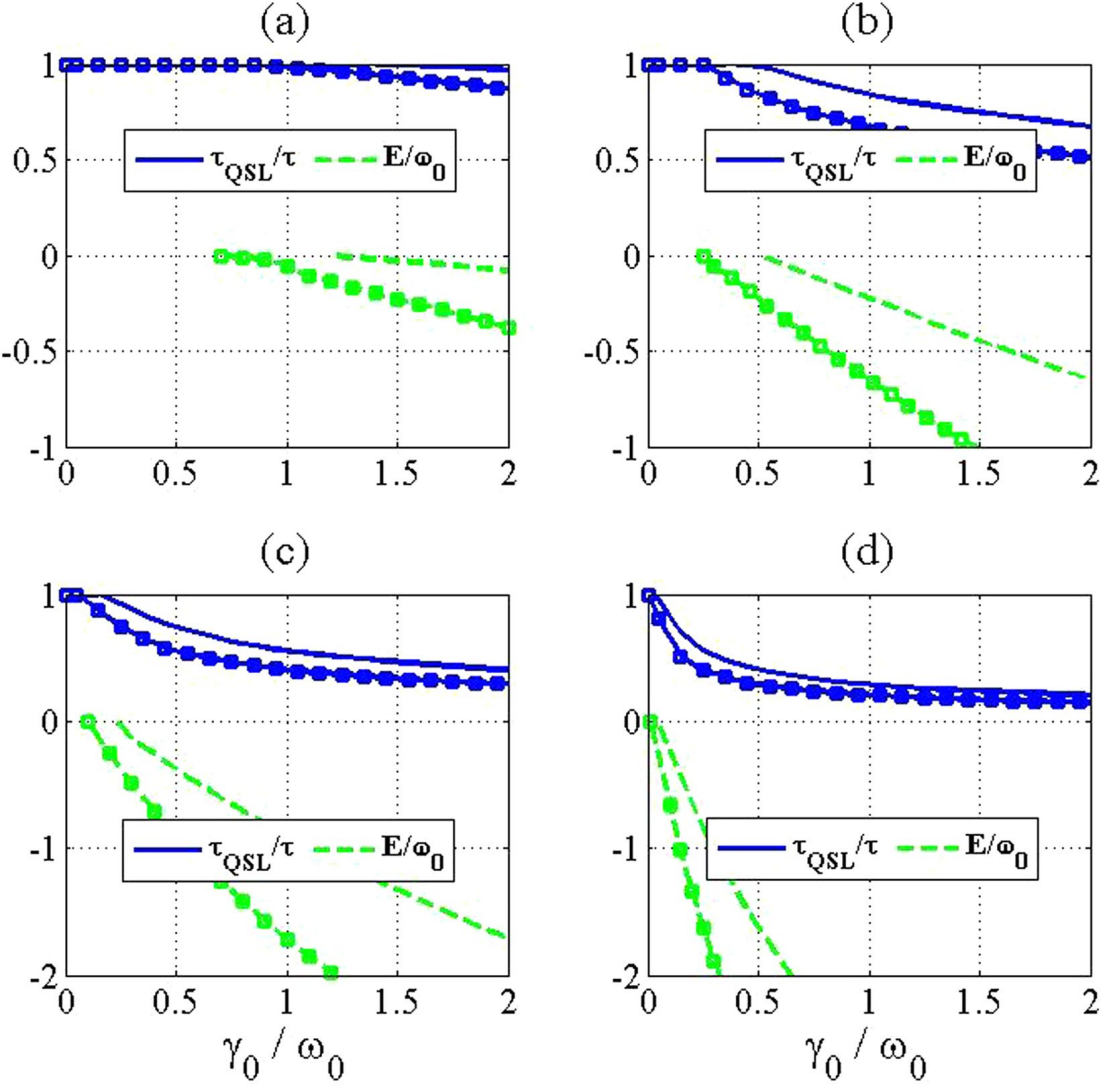
The QSL time (blue solid line) and non-Markovianity (red dashed line) for a V-type three level system in terms of the coupling strength ${\gamma }_{0}/{\omega }_{0}$ and with different numbers of atoms in the reservoir as (**a**) *N* = 1, (**b**) *N* = 3, (**c**) *N* = 8 and (**d**) *N* = 30. The values of the used parameters are $\lambda =2$ (in units of ${\omega }_{0}$) and $\tau =5$ (in units of ${\omega }_{0}^{-1}$). Also, lines with the square marks are plotted for *θ* = 1 and without the marks are plotted for *θ* = 0.

For more investigation of this interesting finding, we proceed further by considering a V-type three-level atom in the presence of similar and additional atoms. In this regard, the ratio ${\tau }_{{\rm{QSL}}}/\tau$ with the non-Markovianity of the V-type three-level atom has been presented in Fig. 4 and with the negative energy spectrum of the total Hamiltonian in Eq. (8), has been presented in Fig. 5. In addition to the previous observations that are also seen for the model considered here, we find that the speedup phenomenon is occurred faster by increasing the SGI parameter. Also, it is clear that increasing the SGI parameter leads to improvement of degree of boundedness, which in turns causes the speedup of the evolution. Consequently, these results reveal that the appearance of system-reservoir bound state with a higher degree of boundedness ensures the enhancement in the intrinsic speed of evolution irrespective of non-Markovianity of the system.

## Conclusion

In conclusion, the mechanism of quantum speedup for a two-level system and a V-type three-level system has been studied in the presence of additional systems from two perspectives: non-Markovianity and formation of a system-environment bound state. We have demonstrated that although non-Markovianity governs the quantum speedup in the absence of additional systems, it is neither necessary nor sufficient to speed up the quantum evolution when the environment becomes complex in the presence of additional systems. Also, it has been revealed that the bound state of the whole system plays a deterministic role in quantum speedup of the considered systems. In other words, providing bound states with more negative energy can lead to greater speed of quantum evolution. Our results may open new perspectives for acceleration of evolution through engineering the formation of a bound state.

## Methods

### Dynamics of a *N* two-level system immersed in a common reservoir

Here, we want to derive the dynamics of an open two-level atomic system in the presence of ($N-1$) additional systems which are immersed in a common reservoir^30^. In this regard, we obtain the exact dynamics of a system consisting of $N$ non-interacting two-level atoms in a common reservoir as considered in case I. For simplicity, we work in the interaction picture

24 $$i\frac{d}{dt}|\psi (t){\rangle }_{I}={H}_{I}(t)|\psi (t){\rangle }_{I},$$

where the Hamiltonian in this picture is given by

25 $${H}_{I}(t)=\mathop{\sum }\limits_{l=1}^{N}\,\sum _{k}\,({g}_{k}{\sigma }_{l}^{+}{b}_{k}{e}^{i({\omega }_{0}-{\omega }_{k})t}+{g}_{k}^{\ast }{\sigma }_{l}^{-}{b}_{k}^{\dagger }{e}^{-i({\omega }_{0}-{\omega }_{k})t}),$$

and

26 $$|\psi (t){\rangle }_{I}={\alpha }_{0}(t)|0{\rangle }_{S}|0{\rangle }_{E}+\mathop{\sum }\limits_{l=1}^{N}\,{\tilde{\alpha }}_{l}(t)|l{\rangle }_{S}|0{\rangle }_{E}+\sum _{k}\,{\tilde{\beta }}_{k}(t)|0{\rangle }_{S}|{1}_{k}{\rangle }_{E},$$

with ${\tilde{\alpha }}_{l}(t)={e}^{i{\omega }_{0}t}{\alpha }_{l}(t)$ and ${\tilde{\beta }}_{k}(t)={e}^{i{\omega }_{k}t}{\beta }_{k}(t)$. Substituting Eqs (25) and (26) into Eq. (24) gives the following differential equations as

27 $$\begin{array}{rcl}\frac{d{\tilde{\alpha }}_{l}(t)}{dt} & = & -i\,\sum _{k}\,{g}_{k}{\tilde{\beta }}_{k}(t){e}^{i({\omega }_{0}-{\omega }_{k})t},\\ \frac{d{\tilde{\beta }}_{k}(t)}{dt} & = & -i\,\mathop{\sum }\limits_{l=1}^{N}\,{g}_{k}^{\ast }{\tilde{\alpha }}_{l}(t){e}^{-i({\omega }_{0}-{\omega }_{k})t},\end{array}$$

with $l=1,2,\ldots ,N$. It is clear that ${H}_{I}(t)|0{\rangle }_{S}\otimes |0{\rangle }_{E}=0$, so ${\alpha }_{0}(t)={\alpha }_{0}(0)={\alpha }_{0}$. Then, integrating the last equation of Eq. (27) and substituting it into the others gives

28 $$\frac{d{\tilde{\alpha }}_{l}(t)}{dt}=-\,{\int }_{0}^{t}\,f(t-t^{\prime} )\,\mathop{\sum }\limits_{u=1}^{N}\,{\tilde{\alpha }}_{u}(t)dt^{\prime} ,$$

where the integration of $J(\omega ){e}^{i({\omega }_{0}-\omega )(t-t^{\prime} )}$ with respect to $\omega$ leads to the correlation function $f(t-t^{\prime} )$ as

29 $$f(t-t^{\prime} )={\int }^{}\,d\omega J(\omega ){e}^{i({\omega }_{0}-\omega )(t-t^{\prime} )}.$$

It is common to use the Lorentzian function as the spectral density of the reservoir. So, we take it as follows

30 $$J(\omega )=\frac{1}{2\pi }\frac{{\gamma }_{0}{\lambda }^{2}}{{(\omega -{\omega }_{0})}^{2}+{\lambda }^{2}},$$

where ${\omega }_{0}$ is the central frequency of the reservoir, the parameter *λ* defines the spectral width of the coupling and *γ*_0_ is the coupling strength. Therefore, by considering the spectral density $J(\omega )$ given by Eq. (30) and using the Laplace transform, the exact solutions of the probability amplitudes ${\tilde{\alpha }}_{l}(t)$ can be obtained as^30^

31 $$\begin{array}{rcl}{\tilde{\alpha }}_{l}(t) & = & {e}^{-\lambda t/2}(\cosh (\frac{Dt}{2})+\frac{\lambda }{D}\,\sinh (\frac{Dt}{2})){\tilde{\alpha }}_{l}(0)\\  &  & +\,(\frac{(N-1){\tilde{\alpha }}_{l}(0)-{\sum }_{l^{\prime} \ne l}^{N}\,{\tilde{\alpha }}_{l^{\prime} }(0)}{N})\\  &  & \times \,(1-{e}^{-\lambda t/2}(\cosh (\frac{Dt}{2})+\frac{\lambda }{D}\,\sinh (\frac{Dt}{2}))),\end{array}$$

where $D=\sqrt{{\lambda }^{2}-2{\gamma }_{0}\lambda N}$. Also, by considering the 1th atom as our main concern of system ($l=1$) and assuming the initial condition as ${\tilde{\alpha }}_{l^{\prime} }(0)=0$ for $l^{\prime} \ne 1$, Eq. (31) is reduced to

32 $${\tilde{\alpha }}_{1}(t)=(\frac{N-1}{N}+\frac{{e}^{-\lambda t/2}}{N}(\cosh (\frac{Dt}{2})+\frac{\lambda }{D}\,\sinh (\frac{Dt}{2}))){\tilde{\alpha }}_{l}(0).$$

Moreover, tracing over the reservoir and the ($N-1$) additional atoms results in an explicit form for the reduced density operator of the 1st atom in the basis of $\{|e\rangle ,|g\rangle \}$ as follows

33 $${\rho }_{1}(t)=(\begin{array}{cc}|{\tilde{\alpha }}_{1}(t){|}^{2} & {\alpha }_{0}^{\ast }{\tilde{\alpha }}_{1}(t)\\ {\alpha }_{0}{\tilde{\alpha }}_{1}{(t)}^{\ast } & 1-|{\tilde{\alpha }}_{1}(t){|}^{2}\end{array}).$$

### Dynamics of *N* V-type three-level system immersed in a common reservoir

In the following, we derive the dynamics of an open V-type three-level atomic system in the presence of ($N-1$) additional systems^31^. To this aim, we obtain the exact dynamics of a system consisting of $N$ non-interacting V-type three-level atoms in a common ${\tilde{\nu }}_{l}^{B}$ reservoir as considered in case II. According to Schrödinger equation in the interaction picture, we have

34 $$i\frac{d}{dt}|\varphi (t){\rangle }_{I}={H}_{I}^{^{\prime} }(t)|\varphi (t){\rangle }_{I},$$

where

35 $${H^{\prime} }_{I}(t)=\mathop{\sum }\limits_{l=1}^{N}\,\sum _{m=A,B}\,\sum _{k}\,({g}_{mk}{\sigma }_{m}^{l+}{b}_{k}{e}^{i({\omega }_{m}-{\omega }_{k})t}+{g}_{mk}^{\ast }{\sigma }_{m}^{l-}{b}_{k}^{\dagger }{e}^{-i({\omega }_{m}-{\omega }_{k})t}),$$

and

36 $$\begin{array}{rcl}|\varphi (t){\rangle }_{I} & = & {\nu }_{0}(t)|0{\rangle }_{S}\otimes |0{\rangle }_{E}+\mathop{\sum }\limits_{l=1}^{N}\,(\tilde{{\nu }_{l}^{A}}(t)|{A}_{l}\rangle +\tilde{{\nu }_{l}^{B}}(t)|{B}_{l}\rangle {)}_{S}\\  &  & \otimes \,|0{\rangle }_{E}+\sum _{k}\,\tilde{{\eta }_{k}}(t)|0{\rangle }_{S}|{1}_{k}{\rangle }_{E},\end{array}$$

with $\tilde{{\nu }_{l}^{A}}(t)={e}^{i{\omega }_{A}t}{\nu }_{l}^{A}(t)$, $\tilde{{\nu }_{l}^{B}}(t)={e}^{i{\omega }_{B}t}{\nu }_{l}^{B}(t)$ and $\tilde{{\eta }_{k}}(t)={e}^{i{\omega }_{k}t}{\eta }_{k}(t)$. It can be easily seen that ${H^{\prime} }_{I}(t)|0{\rangle }_{S}\otimes |0{\rangle }_{E}=0$, then ${\nu }_{0}(t)={\nu }_{0}(0)={\nu }_{0}$, while by substituting Eqs (35) and (36) into Eq. (34), we have the following differential equations

37 $$\begin{array}{l}\frac{d\tilde{{\nu }_{l}^{A}}(t)}{dt}=-\,i\,\sum _{k}\,{g}_{Ak}{e}^{i({\omega }_{A}-{\omega }_{k})t}\tilde{{\eta }_{k}}(t),\\ \frac{d\tilde{{\nu }_{l}^{B}}(t)}{dt}=-\,i\,\sum _{k}\,{g}_{Bk}{e}^{i({\omega }_{B}-{\omega }_{k})t}\tilde{{\eta }_{k}}(t),\\ \frac{d\tilde{{\eta }_{k}}(t)}{dt}=-\,i\,\sum _{m=A,B}\,{g}_{mk}^{\ast }{e}^{-i({\omega }_{m}-{\omega }_{k})t}\,\mathop{\sum }\limits_{l=1}^{N}\,\tilde{{\nu }_{l}^{m}}(t).\end{array}$$

Since it is important for us to obtain the coefficients $\tilde{{\nu }_{l}^{A,B}}$, so we integrate the last differential equation in (37) and substitute it in the rest of differential equations. As a result, we obtain the following set of closed integro-differential equations

38 $$\frac{d\tilde{{\nu }_{l}^{m}}(t)}{dt}=-\,\sum _{n=A,B}\,{\int }_{0}^{t}\,{f}_{mn}(t-t^{\prime} )\,\mathop{\sum }\limits_{j=1}^{N}\,\tilde{{\nu }_{j}^{n}}(t^{\prime} )dt^{\prime} ,\,m=A,B.$$

It should be noted that the kernels in Eq. (38) can be expressed in terms of spectral density $J(\omega )$ as follows

39 $${f}_{mn}(t-t^{\prime} )={\int }_{0}^{t}\,d\omega {J}_{mn}(\omega ){e}^{i({\omega }_{m}-\omega )t-i({\omega }_{n}-\omega )t^{\prime} },$$

and $J(\omega )$ is chosen as Lorentzian distribution

40 $${J}_{mn}(\omega )=\frac{1}{2\pi }\frac{{\gamma }_{mn}{\lambda }^{2}}{{({\omega }_{0}-\omega )}^{2}+{\lambda }^{2}},$$

where ${\omega }_{0}$ and *λ* are respectively the center frequency of the structured reservoir and the spectral width of the coupling. Also, ${\gamma }_{mm}={\gamma }_{m}$ show the relaxation rates of the two upper excited levels. And, ${\gamma }_{mn}=\sqrt{{\gamma }_{m}{\gamma }_{n}}\theta$, provided that $m\ne n$, stand for the SGI between existing transitions in each atom. It is worth to note that the parameter $\theta$ depends on the relative angle between two dipole moment elements of the mentioned transitions. While $\theta =0$, indicating no SGI between the two transitions, reflects the perpendicularity of the dipole moments of transitions to each other, on the other hand, $\theta =1$ reflects that the two dipole moments are parallel, verifying the strongest SGI between the transitions. The Laplace transform of Eq. (38) results in

41 $$p\tilde{{\nu }_{l}^{m}}(p)-\tilde{{\nu }_{l}^{B}}(0)=-\,\sum _{n=A,B}\, {\mathcal L} \{{f}_{mn}(t)\}\,\mathop{\sum }\limits_{j=1}^{N}\,\tilde{{\nu }_{j}^{n}}(p),\,m=A,B.$$

Here, $\tilde{{\nu }_{l}^{m}}(p)= {\mathcal L} \{\tilde{{\nu }_{l}^{m}}(t)\}={\int }_{0}^{\infty }\,\tilde{{\nu }_{l}^{m}}(t){e}^{-pt}dt$, is the Laplace transform of $\tilde{{\nu }_{l}^{m}}(t)$. According to Eq. (41), it is obvious that the following relation between the coefficients is found

42 $$p\tilde{{\nu }_{l}^{m}}(p)-\tilde{{\nu }_{l}^{m}}(0)=\cdots =p\tilde{{\nu }_{l}^{m}}(p)-\tilde{{\nu }_{l}^{m}}(0)=\cdots =p\tilde{{\nu }_{N}^{m}}(p)-\tilde{{\nu }_{N}^{m}}(0).$$

Now, writing the coefficients $\tilde{{\nu }_{j}^{m}}(p)$ ($j\ne l$) in terms of $\tilde{{\nu }_{l}^{m}}(p)$ and inserting them into the Eq. (41), leads to the following equation

43 $$p\tilde{{\nu }_{l}^{m}}(p)-\tilde{{\nu }_{l}^{m}}(0)=-\,\sum _{n=A,B}\, {\mathcal L} \{{f}_{mn}(t)\}(N\tilde{{\nu }_{l}^{n}}(p)+\frac{1}{p}\,\mathop{\sum }\limits_{j\ne l}^{N}\,(\tilde{{\nu }_{j}^{n}}(0)-\tilde{{\nu }_{l}^{n}}(0))).$$

Here, we turn our attention to a special case in which the degeneration of two upper atomic states, and the resonance between the atomic transitions and the central frequency of the reservoir are considered, i.e., ${\omega }_{A}={\omega }_{B}={\omega }_{0}$. Under this consideration, we can assume that ${\gamma }_{A}={\gamma }_{B}={\gamma }_{0}$ and ${\gamma }_{AB}={\gamma }_{BA}={\gamma }_{0}\theta$, so the kernels in Eq. (39) takes the following simple form

44 $$\begin{array}{rcl}{f}_{AA}(t) & = & {f}_{BB}(t)=f(t)={\int }_{0}^{t}\,d\omega J(\omega ){e}^{i({\omega }_{0}-\omega )(t-t^{\prime} )},\\ {f}_{AB}(t) & = & {f}_{BA}(t)=f^{\prime} (t)={\int }_{0}^{t}\,d\omega J^{\prime} (\omega ){e}^{i({\omega }_{0}-\omega )(t-t^{\prime} )},\end{array}$$

where $J^{\prime} (\omega )=\theta J(\omega )$. In the following, we define the new coefficients $\tilde{{\nu }_{l}^{\pm }}(p)=\tilde{{\nu }_{l}^{A}}(p)\pm \tilde{{\nu }_{l}^{B}}(p)$, and rewrite Eq. (43) in terms of them as

45 $$\begin{array}{rcl}p\tilde{{\nu }_{l}^{\pm }}(p)-\tilde{{\nu }_{l}^{\pm }}(0) & = & -[ {\mathcal L} \{f(t)\}\pm  {\mathscr L} \{f^{\prime} (t)\}](N\tilde{{\nu }_{l}^{\pm }}(p)\\  &  & +\,\frac{1}{p}\,\mathop{\sum }\limits_{j\ne l}^{N}\,(\tilde{{\nu }_{j}^{\pm }}(0)-\tilde{{\nu }_{l}^{\pm }}(0))),\end{array}$$

therefore, after taking inverse Laplace transform we get

46 $$\tilde{{\nu }_{l}^{\pm }}(t)={\mathscr{G}}_{\pm }(t)\tilde{{\nu }_{l}^{\pm }}(0)-\frac{1-{\mathscr{G}}_{\pm }(t)}{N}\,\mathop{\sum }\limits_{j\ne l}^{N}\,(\tilde{{\nu }_{j}^{\pm }}(0)-\tilde{{\nu }_{l}^{\pm }}(0)),$$

where ${\mathscr{G}}_{\pm }(t)={e}^{-\lambda t/2}(\cosh (\frac{{D}^{\pm }t}{2})+\frac{\lambda }{{D}^{\pm }}\,\sinh (\frac{{D}^{\pm }t}{2}))$ and ${D}^{\pm }=\sqrt{{\lambda }^{2}-2{\gamma }_{0}(1\pm \theta )\lambda N}$. Finally, it should be noted that we take the first atom ($l=1$) of the studied system as our main concern of the single three-level system and the ($N-1$) remaining atoms as the additional atoms. Hence, tracing over the reservoir and the ($N-1$) additional atoms leads to the explicit form of the reduced density operator of the first atom in the basis of $\{|{A}_{1}\rangle ,|{B}_{1}\rangle ,|{C}_{1}\rangle \}$ as follows

47 $${\rho }_{1}(t)=(\begin{array}{ccc}|\tilde{{\nu }_{1}^{A}}(t){|}^{2} & \tilde{{\nu }_{1}^{A}}(t)\tilde{{\nu }_{1}^{B}}{(t)}^{\ast } & \tilde{{\nu }_{1}^{A}}(t){\nu }_{0}^{\ast }\\ \tilde{{\nu }_{1}^{B}}(t)\tilde{{\nu }_{1}^{A}}{(t)}^{\ast } & |\tilde{{\nu }_{1}^{B}}(t){|}^{2} & \tilde{{\nu }_{1}^{B}}(t){\nu }_{0}^{\ast }\\ {\nu }_{0}\tilde{{\nu }_{1}^{A}}{(t)}^{\ast } & {\nu }_{0}\tilde{{\nu }_{1}^{B}}{(t)}^{\ast } & 1-|\tilde{{\nu }_{1}^{A}}(t){|}^{2}-|\tilde{{\nu }_{1}^{B}}(t){|}^{2}\end{array}).$$
